# Adverse Drug Reactions Relevant to Drug Resistance and Ineffectiveness Associated with Meropenem, Linezolid, and Colistin: An Analysis Based on Spontaneous Reports from the European Pharmacovigilance Database

**DOI:** 10.3390/antibiotics12050918

**Published:** 2023-05-16

**Authors:** Bogdan Ioan Vintila, Anca Maria Arseniu, Anca Butuca, Mihai Sava, Victoria Bîrluțiu, Luca Liviu Rus, Dan Damian Axente, Claudiu Morgovan, Felicia Gabriela Gligor

**Affiliations:** 1 Clinical Surgical Department, Faculty of Medicine, “Lucian Blaga” University of Sibiu, 550169 Sibiu, Romania; bogdan.vintila@ulbsibiu.ro (B.I.V.); mihai.sava@ulbsibiu.ro (M.S.); 2 County Clinical Emergency Hospital, 550245 Sibiu, Romania; victoria.birlutiu@ulbsibiu.ro; 3 Preclinical Department, Faculty of Medicine, “Lucian Blaga” University of Sibiu, 550169 Sibiu, Romania; anca.butuca@ulbsibiu.ro (A.B.); liviu.rus@ulbsibiu.ro (L.L.R.); claudiu.morgovan@ulbsibiu.ro (C.M.); felicia.gligor@ulbsibiu.ro (F.G.G.); 4 Clinical Medical Department, Faculty of Medicine, “Lucian Blaga” University of Sibiu, 550169 Sibiu, Romania; 5 Fifth Surgical Clinic, “Iuliu Hatieganu” University of Medicine and Pharmacy, 400012 Cluj-Napoca, Romania; damian.axente@umfcluj.ro

**Keywords:** antibiotic treatment failure, meropenem, linezolid, colistin, antimicrobial resistance, EudraVigilance, pharmacovigilance

## Abstract

Antimicrobial resistance is considered one of the major threats to public health and is an important factor that influences the patient’s outcome in the intensive care unit. Pharmacovigilance can help raise awareness of potential drug resistance (DR) or ineffectiveness (DI) through adverse drug reaction reports that are submitted to different spontaneous reporting systems. Based on spontaneous Individual Case Safety Reports from EudraVigilance, we conducted a descriptive analysis of adverse drug reactions associated with meropenem, colistin, and linezolid, with a focus on DR and DI. Of the total adverse drug reactions (ADRs) reported for each analyzed antibiotic by 31 December 2022, between 2.38–8.42% and 4.15–10.14% of the reports were related to DR and DI, respectively. A disproportionality analysis was conducted to evaluate the frequency of reporting adverse drug reactions relevant to the DR and DI of the analyzed antibiotics compared to other antimicrobials. Based on the analysis of the collected data, this study underlines the importance of post-marketing drug safety monitoring in raising a warning signal regarding antimicrobial resistance, thereby potentially contributing to the reduction in antibiotic treatment failure in an intensive care setting.

## 1. Introduction

Nowadays, antimicrobial resistance (AMR) is one of the biggest threats to public health. The inadequate and irrational use of antibiotics is one of the main factors responsible for the development of AMR. In critically ill patients, the correct antibiotic regimen is very important because several physiological manifestations of a critical illness may change the pharmacokinetic behavior of administered drugs [1]. Pharmacokinetic modifications in critically ill patients are present without exception, from absorption, distribution, and metabolism to the process of excretion [2]. Thus, in the intensive care patient, standard antibiotic doses are expected to result in over- or underexposure to drugs, mainly due to changes in the volume of distribution and clearance [3].

According to their solubility, antibiotics can be categorized as hydrophilic and lipophilic, which can help to describe possible altered pharmacokinetics that can be caused by the pathophysiological changes common to critical illnesses [4]. Hydrophilic antibiotics are mostly affected by the pathophysiological changes observed in critically ill patients, with increased volumes of distribution and altered drug clearance; on the other hand, lipophilic antibiotics have a lesser volume of distribution alterations but may develop altered drug clearances [5]. 

It is widely known that antimicrobial resistance is a natural phenomenon and our actions are not the sole cause of it [6]. Factors that contribute to the emergence of this process and are related to human behavior include poor community hygiene, antibiotic accumulation in the environment and the food products consumed, and clinical settings with inadequate infection control measures, as well as overuse and misuse of antimicrobials [7,8]. 

An example of misuse of antimicrobials is the suboptimal exposure at the routine dose of an antibiotic to a critically ill patient [9]. Due to their altered physiology, the plasmatic concentration is suboptimal for this patient population [10]. In the case of subtherapeutic dosing, antibiotic resistance may develop and/or therapeutic failure may occur [11]. This issue can be addressed by a personalized approach of the critically ill patient to contain resistance spread [9].

Among patients treated in the intensive care unit (ICU), there is a high rate of severe infections that is associated with high morbidity and mortality [12]. Thereby, it is estimated that 71% of the patients admitted to the ICU are under antibiotic treatment [13]. Meropenem (MER), linezolid (LIN), and colistin (COL) are commonly used to treat infections produced by multi-drug-resistant microorganisms that are encountered in ICU patients [14,15,16,17] and will be briefly reviewed.

Carbapenems are a class of antibacterial drugs with a broad spectrum of activity and are considered the first-line therapy for drug-resistant infections caused by gram-positive or gram-negative pathogens [18]. Moreover, their toxicity is considered low compared to other antimicrobials and they are a safer alternative to other last-line drugs used to treat bacterial infections [19]. Carbapenems alter the bacterial cell wall biosynthesis through the inhibition of penicillin-binding proteins (PBPs), leading to cell lysis and cell death [20]. Resistance to carbapenems can occur through PBP alteration, the production of carbapenemases, the overexpression of efflux pumps, or reduced porin-mediated cellular permeability [21]. 

Linezolid belongs to the class of oxazolidinone antibiotics and is authorized for the treatment of infections caused by gram-positive pathogens, such as bacterial pneumonia, skin and soft tissue infections, and vancomycin-resistant *Enterococcus faecium* infections [15,22]. Linezolid inhibits bacterial protein synthesis by binding directly to the 50S ribosomal subunit, preventing the formation of a functional 70S initiation complex [23]. Small alterations to the binding site on the ribosome are responsible for the development of resistance to linezolid, but due to its unique mechanism of action, cross-resistance with other protein synthesis inhibitors is unlikely [24,25]. 

Polymyxins are a class of antibiotics consisting of polymyxin B and polymyxin E, also known as colistin. Even though they were introduced into clinical practice in the 1950s, interest in these antibiotics has been regained in the last two decades with the emergence of multi-drug-resistant gram-negative bacteria that do not respond to other clinically available antibacterial drugs [26]. A frequent adverse reaction associated with the systemic use of polymyxins is nephrotoxicity [27]. Many risk factors for polymyxin nephrotoxicity have been documented in the literature, such as the duration of therapy, daily dose, and cumulative dose, concomitant use of other nephrotoxic drugs, patient age, the presence of other renal dysfunctions or disease, obesity, hypertension, etc. [28]. Polymyxins act by disrupting the integrity of the outer cell membrane as a result of binding to the negatively charged lipid A moiety of lipopolysaccharide (LPS) changes in the structure of LPS lead to the development of acquired resistance to polymyxin in gram-negative bacteria [29,30]. Moreover, some gram-negative bacteria, such as *Proteus mirabilis*, *Helicobacter pylori*, *Moraxella catarrhalis*, *Providencia* spp., *Serratia marcescens*, etc., are known to possess intrinsic resistance to polymyxins [31]. 

More than 170 countries and territories are part of the World Health Organization Programme for International Drug Monitoring [32], and the pharmacovigilance network across these full or associate member states is a useful complementary method to raise awareness of suspected antimicrobial resistance or inappropriate use of antibiotics [33]. A study conducted by Truong et al. revealed that drug resistance (DR) and drug ineffectiveness (DI) are among the ten most common serious adverse drug reactions (ADRs) reported for colistin in the U.S. Food and Drug Administration Adverse Event Reporting System database [34].

The increase in the number of ADR reports related to drug resistance or the ineffectiveness of antibiotics can be seen as a warning signal of the increasing phenomenon of antimicrobial resistance [33]. 

This study aimed to perform a descriptive analysis of ADRs reported for meropenem, colistin, and linezolid, based on spontaneous reports from EudraVigilance (EV), the European database of suspected adverse drug reaction reports. The reporting frequency of ADRs relevant to drug resistance and ineffectiveness was evaluated. Furthermore, a disproportionality analysis was conducted to evaluate the reporting rate of the above-mentioned categories of ADRs compared to other antibiotics or antifungals.

## 2. Results

### 2.1. Descriptive Analysis

We performed a descriptive analysis of Individual Case Safety Reports (ICSRs) submitted in EV associated with COL, MER, and LIN, taking into consideration characteristics such as age, sex, geographical origin, and category of reporters. Additionally, the ICSRs containing preferred terms related to DR (3 PTs) and DI (9 PTs) reported for COL, LIN, and MER were analyzed further, and the results are presented below.

#### 2.1.1. Characteristics of Individual Case Safety Reports

**Figure 1:**
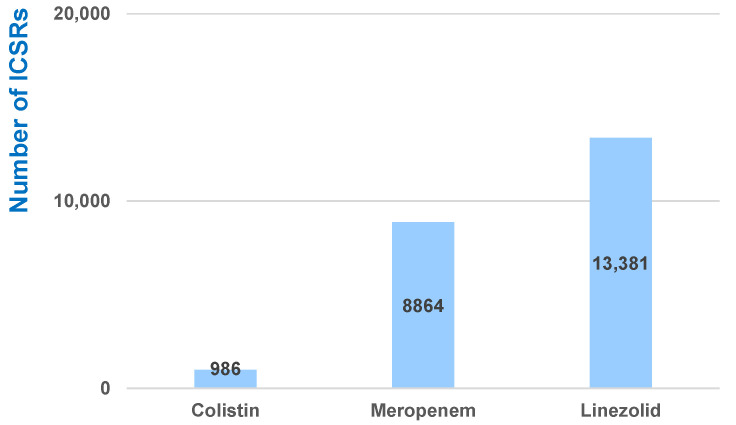
Total ICSRs reported in EV.

Until 31 December 2022, a total of 13,381 ICSRs were submitted to EV for LIN, followed by 8864 and 986 reports for MER and COL, respectively (Figure 1). 

**Figure 2:**
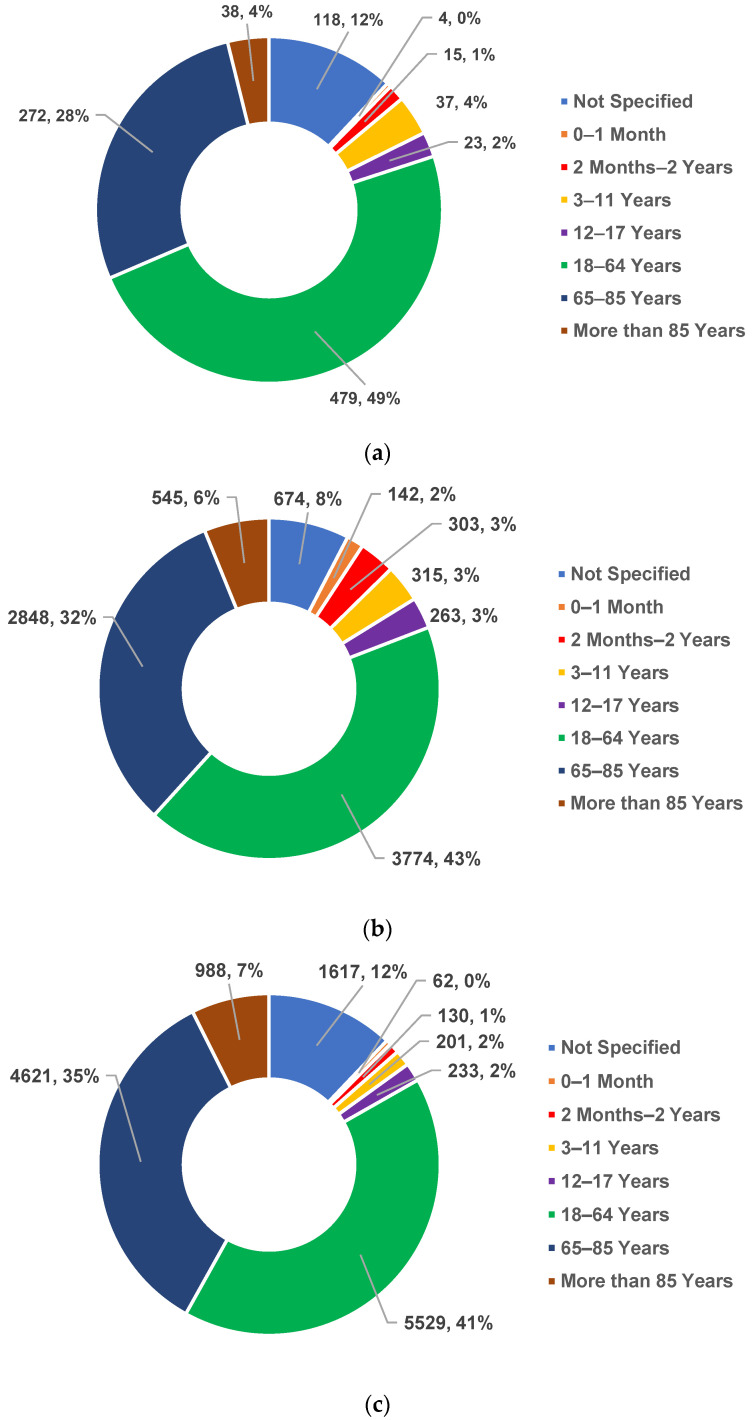
Distribution of total ICSRs by patients’ age: (**a**) colistin; (**b**) meropenem; (**c**) linezolid.

The distribution of these ICSRs according to patient age is shown in Figure 2. According to the European Medicines Agency (EMA) rules, the age groups are 0–1 month, 2 months–2 years, 3–11 years, 12–17 years, 18–64 years, 65–85 years, more than 85 years, and not specified. ADRs were reported in every age group for all three antibiotics, but most frequently they were registered in patients aged between 18–64 years (COL—49%; MER—43%; LIN—41%) and 65–85 years (COL—28%; MER—32%; LIN—35%).

**Figure 3:**
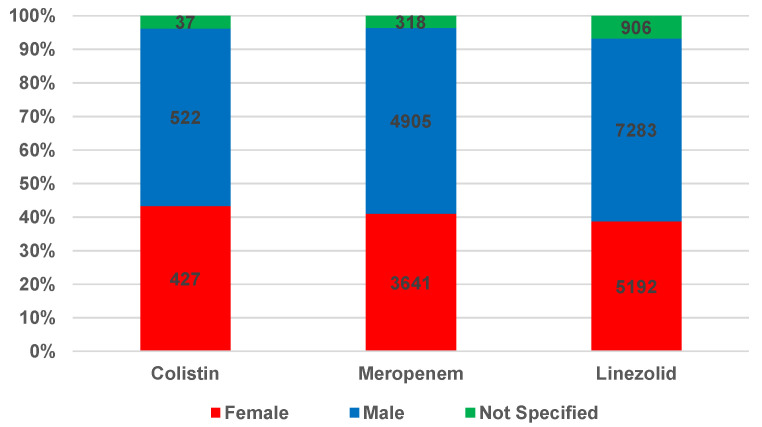
Distribution of total ICSRs by patients’ sex.

Another criterion used in the present descriptive analysis was patient sex (female, male, or not specified). The distribution of ICSRs related to patients’ sex is represented in Figure 3. According to the data displayed in Figure 3, the most frequent reports are in the male group (MER—55.3%, LIN—54.4%, and COL—52.9%). In the female group, the ADR reports are distributed as follows: 43.3%—COL, 41.1%—MER, and 38.8%—LIN.

**Figure 4:**
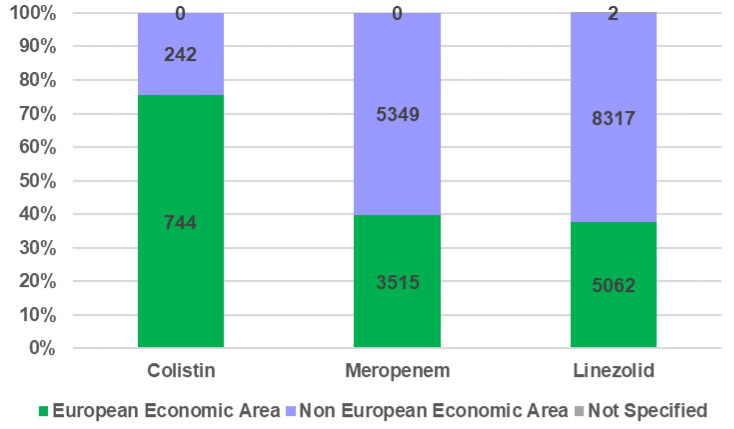
Distribution of total ICSRs by geographical origin of the person who performed the reporting.

The distribution of ICSRs according to the geographical origin of patients (European Economic Area (EEA) and non-EEA) is illustrated in Figure 4. The geographical origin was not specified on only two reports. A total of 75% of the total ICSRs registered for colistin are from EEA, 40% for MER, and 38% for LIN.

**Figure 5:**
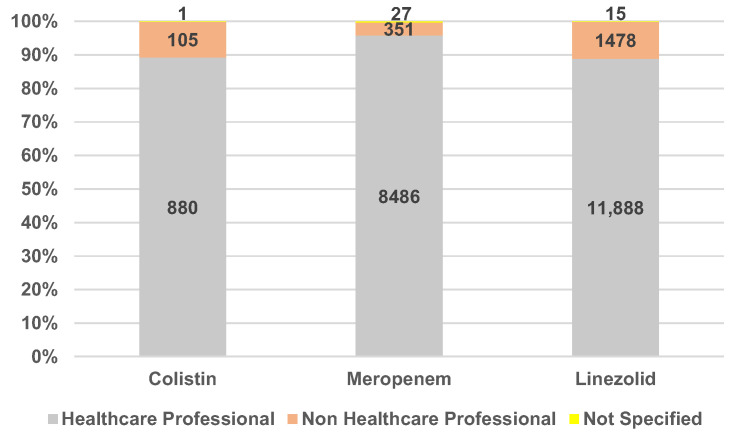
Distribution of total ICSRs by the person who performed the reporting.

Figure 5 presents the distribution of total ICSRs by reporter group. There are two categories of reporters: healthcare professionals and non-healthcare professionals. In a few ADR reports, the reporter group was not specified. Regarding the distribution by reporter group, it was observed that the most frequent ICSRs were registered by healthcare professionals (89%—COL and LIN and 96%—MER).

#### 2.1.2. Drug Resistance and Drug Ineffectiveness

**Figure 6:**
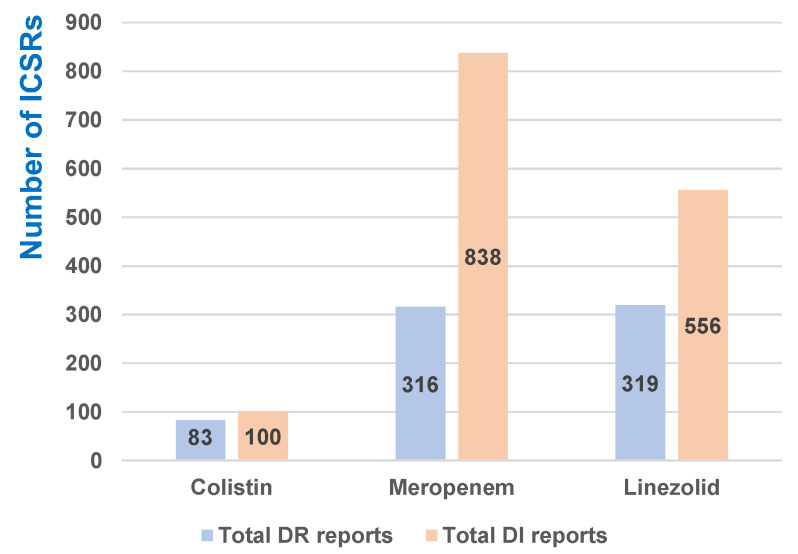
Distribution of total ICSRs by analyzed category of ADRs.

Figure 6 presents the total reports included in the DR and DI categories for all three antibiotics. Of the total ICSRs reported for each drug (Figure 1), the reports related to both categories of ADRs (DR and DI) were most frequently registered for COL (DR—8.42%, n = 83 and DI—10.14%, n = 100). Additionally, the percentage of reports related to MER ineffectiveness is high (9.45%, n = 838). All PTs referring to resistance to MER (3.56%, n = 316) and LIN (2.38%, n = 319) are reported to be less than COL.

**Figure 7:**
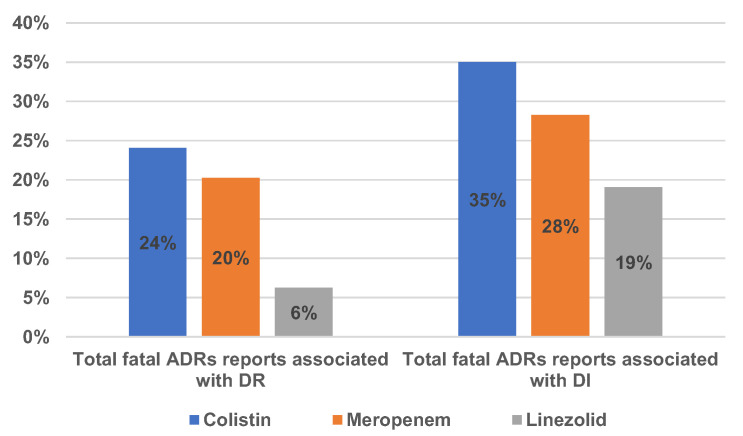
The percentage of fatal ADRs reported among all reports associated with DR and DI.

The distribution of reported fatal ADRs associated with DR and DI is shown in Figure 7. In the DR category, the percentage of fatal ADRs among all reports was 24% for COL, 20% for MER, and 6% for LIN. Higher percentages, but in the same order, were noticed for the DI category: 35%—COL, 28%—MER, and 19%—LIN.

**Figure 8:**
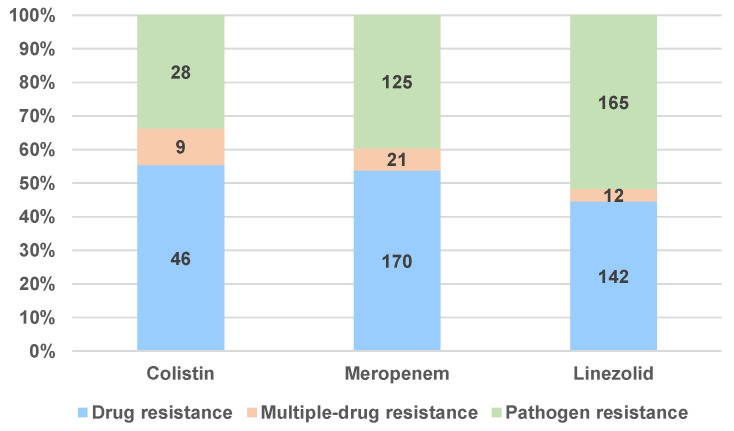
Distribution of total DR reports by type of PTs.

**Figure 9:**
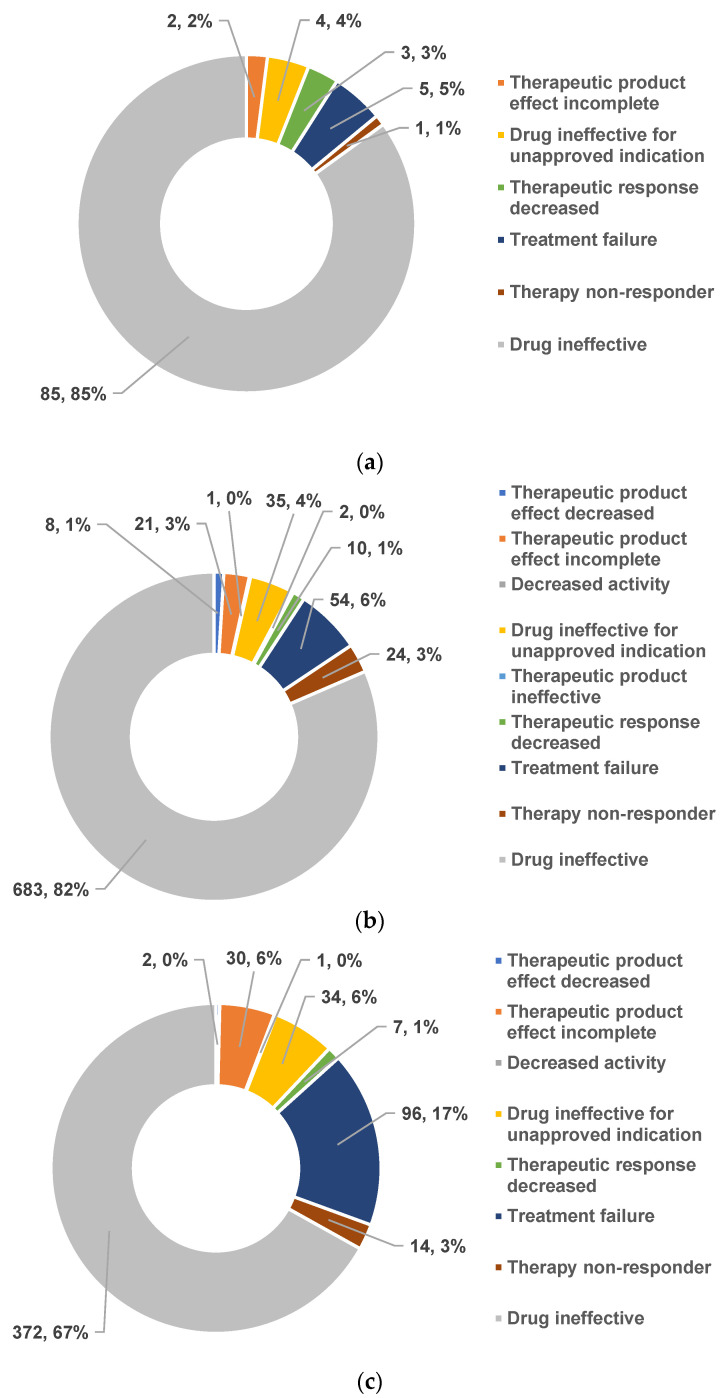
Distribution of DI reports by preferred terms: (**a**)—colistin; (**b**)—meropenem; (**c**)—linezolid.

Figure 8 and Figure 9a–c present the distribution of the ADR reports associated with DR and DI, taking into account the number of ICSRs reported for each PT (three PTs for DR and nine PTs for DI). In the DR category the most used PT for reporting was “Drug resistance” (COL: n = 46, 55.42%, MER: n = 170, 53.80%, LIN: n = 142, 44.51%), followed by “Pathogen resistance”, which was found in 165 reports (51.72%) for LIN, 125 reports (39.56%) for MER, and 28 reports (33.73%) for COL. Referring to the “Multi-drug resistance” preferred term, the percentages are lower: COL—10.84% (n = 9), MER—6.65% (n = 21), and LIN—3.76% (n = 12) (Figure 8).

The distribution of ADR reports containing PTs relevant to DI is presented in Figure 9a–c. The most frequently used PT was “drug ineffective” (COL: n = 85, 85%, MER: n = 683, 81.5%, and LIN: n = 372, 66.91%), followed by “treatment failure” (COL: n = 5, 5%, MER: n = 54, 6.44%, and LIN: n = 96, 17.27%) and “drug ineffective for unapproved indication” (COL: n = 4, 4%, MER: n = 35, 4.18%, LIN: n = 34, 6.12%).

### 2.2. Disproportionality Analysis

In order to perform the disproportionality analysis, the reporting odds ratio (ROR) and 95% confidence interval (95% CI) were calculated comparing to other antimicrobial drugs: amphotericin (AMF), caspofungin (CAP), ceftazidime/avibactam (CEF/AVI), fluconazole (FLU), isavuconazole (ISA), moxifloxacin (MOX), piperacillin/tazobactam (PIP/TAZ), tigecycline (TIG), vancomycin (VAN), and voriconazole (VOR).

#### 2.2.1. Drug Resistance

**Figure 10:**
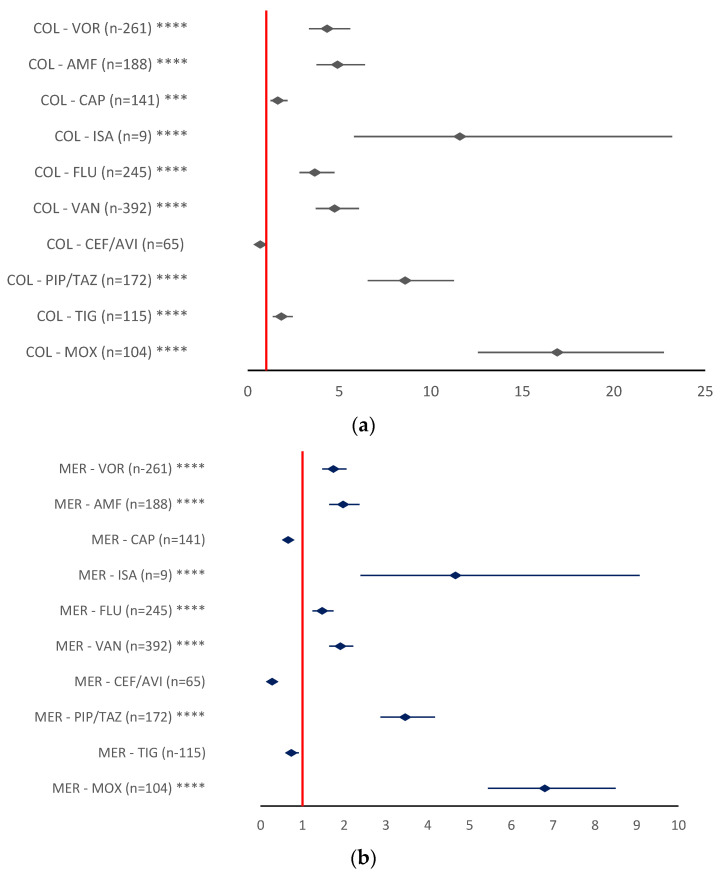

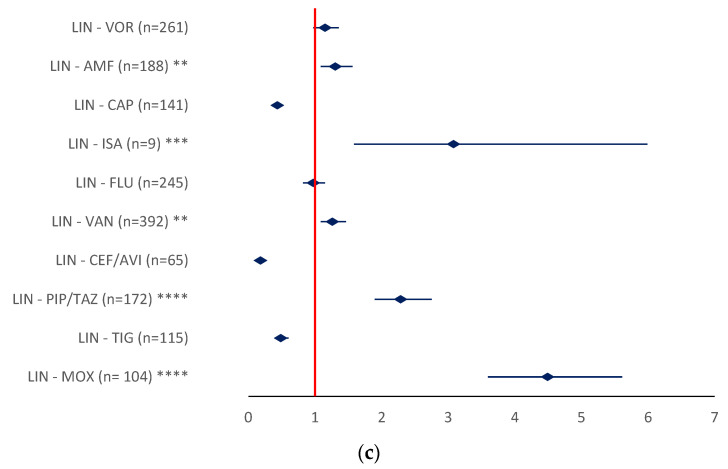
Reporting odds ratio of antibiotics—drug resistance of ADRs: (**a**) colistin; (**b**)—meropenem; (**c**)—linezolid. The disproportionality signal is assumed if a minimum of five ADRs were reported and the ROR is greater than one. AMF—amphotericin; CAP—caspofungin; CEF/AVI—ceftazidime/avibactam; COL—colistin; FLU—fluconazole; ISA—isavuconazole; LIN—linezolid; MER—meropenem; MOX—moxifloxacin; PIP/TAZ—piperacillin/tazobactam; TIG—tigecycline; VAN—vancomycin; VOR—voriconazole; ** *p* ≤ 0.01; *** *p* ≤ 0.001; **** *p* ≤ 0.0001.

Compared to other antibiotics or antifungal drugs (except CEF/AVI—ROR 0.6816, 95% CI 0.4837–0.9604), colistin had a higher reporting probability for all PTs related to DR. The highest reporting probability for the ADRs evaluated was registered when compared to MOX (ROR 16.9090, 95% CI 12.5741–22.7360), ISA (ROR 11.5916, 95% CI 5.7945–23.1885), and PIP/TAZ (ROR 8.6005, 95% CI 6.5629–11.2708) (Figure 10a). 

Meropenem and linezolid showed a higher reporting probability for the AD3Rs relevant for DR when compared to the same three drugs (MOX, PIP/TAZ, and ISA):

- MER-MOX (ROR 6.8006, 95% CI 5.4411–8.4999), MER-PIP/TAZ (ROR 3.4591, 95% CI 2.8675–4.1727), and MER-ISA (ROR 4.662, 95% CI 2.3897–9.0695) (Figure 10b).
- LIN-MOX (ROR 4.927, 95% CI 3.5967–5.6119), LIN-PIP/TAZ (ROR 2.2852, 95%CI 1.8957–2.7546), and LIN-ISA (ROR 3.0799, 95% CI 1.5835–5.9903) (Figure 10c).

When compared to TIG (ROR 0.7352, 95% CI 0.5909–0.9146), CEF/AVI (ROR 0.2741, 95% CI 0.2067–0.3635), and CAP (ROR 0.6576, 95% CI 0.1211–0.8059) a lower reporting probability was found for meropenem (Figure 10b).

Additionally, according to Figure 10c, a lower reporting probability for the ADRs relevant for DR was observed for LIN when compared to TIG (ROR 0.4857, 95% CI 0.3906–0.6038), CEF/AVI (ROR 0.1811, 95% CI 0.1366–0.2400), FLU (ROR 0.9729, 95% CI 0.8220–1.1515), CAP (ROR 0.4344, 95% CI 0.3547–0.5320), and VOR (ROR 1.1505, 95% CI 0.9751–1.3575). 

#### 2.2.2. Drug Ineffectiveness

**Figure 11:**
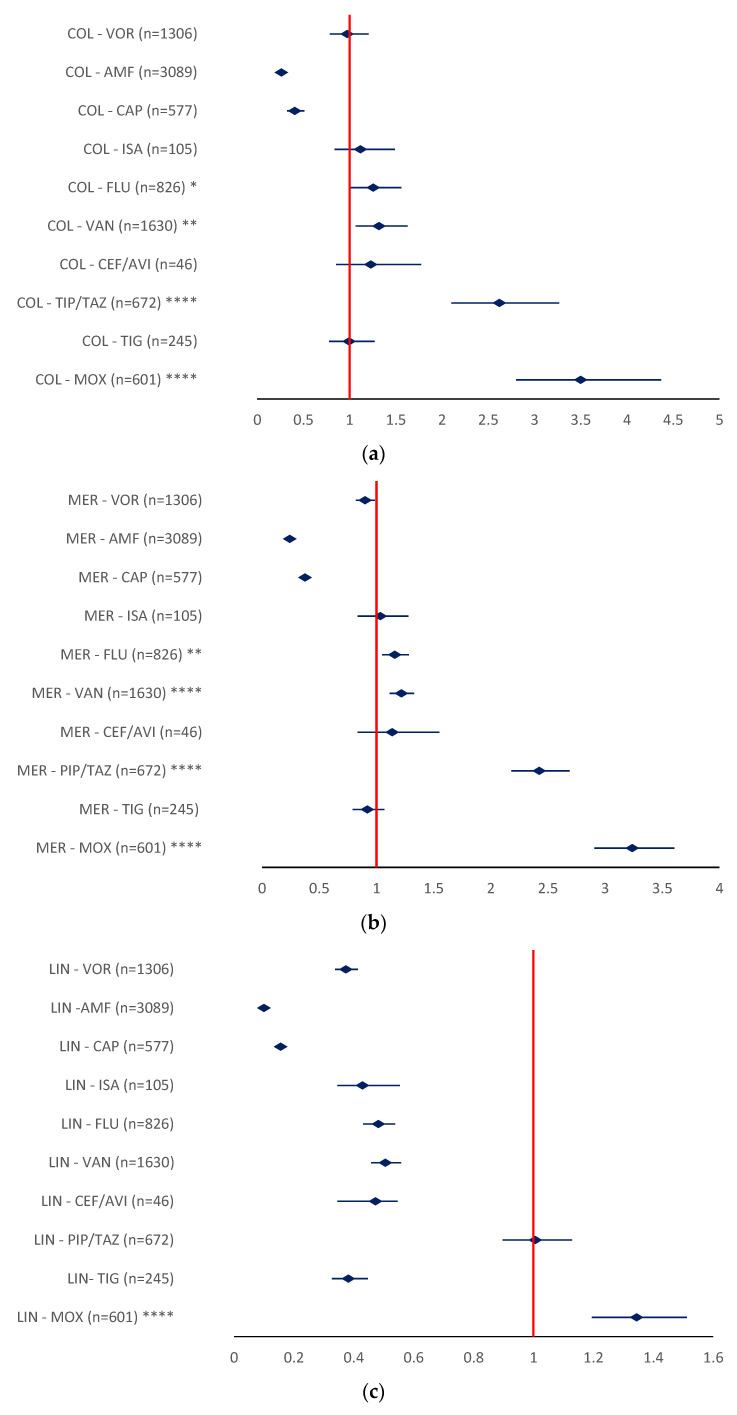
The reporting odds ratio of antibiotics—drug ineffectiveness of ADRs: (**a**) colistin; (**b**)—meropenem; (**c**)—linezolid. The disproportionality signal is assumed if a minimum of five reactions were reported and the ROR is greater than one. AMF—amphotericin; CAP—caspofungin; CEF/AVI—ceftazidime/avibactam; COL—colistin; FLU—fluconazole; ISA—isavuconazole; LIN—linezolid; MER—meropenem; MOX—moxifloxacin; PIP/TAZ—piperacillin/tazobactam; TIG—tigecycline; VAN—vancomycin; VOR—voriconazole; * *p* <0.05; ** *p* ≤ 0.01; **** *p* ≤ 0.0001.

Regarding the ADRs relevant for drug ineffectiveness, a higher probability of reporting was found for colistin when compared to MOX (ROR 3.4996, 95% CI 2.8025–4.3702), PIP/TAZ (ROR 2.6191, 95% CI 2.1004–3.2660), VAN (ROR 1.3158, 95% CI 1.0635–1.6279), and FLU (ROR 1.2542, 95% CI 1.0079–1.5608) (Figure 11a). Meropenem had a higher probability of DI ADR reporting when compared to MOX (ROR 3.2374, 95% CI 2.9060–3.6066), PIP/TAZ (ROR 2.4229, 95% CI 2.1814–2.6911), VAN (ROR 1.2172, 95% CI 1.1154–1.3282), and FLU (ROR 1.1603, 95% CI 1.0492–1.2831) (Figure 11b). 

The calculated ROR for linezolid ineffectiveness was only higher than one when compared to MOX (ROR 1.3442, 95% CI 1.1952–1.5118) (Figure 10c). According to these results, LIN ineffectiveness only showed a higher probability of reporting when compared to MOX. 

## 3. Discussion

The post-marketing surveillance studies play a key role in better characterizing the benefit/risk ratio of a drug. Pharmacovigilance is defined as “the science and activities relating to the detection, assessment, understanding, and prevention of adverse effects or any other possible drug-related problems”, including medication errors, drug ineffectiveness, off-label use, drug abuse, assessment of drug-related mortality, poisoning events, drug interactions, etc. [35]. According to a study conducted by the Upsala Monitoring Centre, terms related to a lack of therapeutic effect were identified as the ninth most frequently reported adverse reaction in VigiBase, the WHO global database of spontaneously reported suspected ADRs [36]. 

The EV database is a collection of suspected ADR reports, coordinated by the EMA [37]. 

The increasing prevalence of the antimicrobial resistance phenomenon together with a lack of progress in the development of new antibiotics represents a growing global health problem. Approximately 50% of prescribed antimicrobial drugs are considered unnecessary, with improper use of antimicrobials being a major problem leading to antimicrobial resistance and subsequently to many ADRs, including the risk of developing difficult-to-treat infections caused by multi-drug-resistant pathogens. Therefore, the growing phenomenon of antimicrobial resistance is leading to the ineffectiveness of antibiotic therapy for certain bacterial infections and subsequently to medical complications, longer hospitalization, and increased mortality [38,39,40].

The AWaRe (Access, Watch, Reserve) classification of antibiotics was developed by the World Health Organization as a tool for antibiotic stewardship, emphasizing the importance of their optimal use to reduce antimicrobial resistance. According to this classification, MER is included in the Watch group, and LIN and COL in the Reserve group [41].

In this study, we analyzed ADR reports associated with COL, LIN, and MER that were submitted to the EV database. By 31 December 2022, a total of 13,381 ADRs were reported for LIN, followed by 8864 reports for MER, and 986 for COL. Multiple reasons can contribute to the higher number of ADRs reported for linezolid, such as increased use, even if it is a relatively new antibiotic; duration of treatment, since it is used long-term; and patient population, since it is primarily used by critically ill patients who are more susceptible to adverse reactions due to the presence of many risk factors [15]. In addition, the off-label use of linezolid, which is quite common, may be a reason for higher risk of ADRs [42].

Based on ICSR distribution according to patient age, we found that all three antibiotics were associated with ADRs, most often occurring in patients 18–64 years of age and 65–85 years old. In the elderly and adult population, the risk of ADR is higher due to pre-existing pathology [43]. An additional explanation would be the increasing age of patients admitted to the ICU [44].

In the present study, a higher frequency of reported ADRs associated with the three analyzed antibiotics was observed in males compared to females. ADRs may develop more frequently in males as a result of differences in pharmacokinetic processes, which may affect drug exposure and ADR development [45]. There may also be an explanation for the distribution of ICSRs in terms of disease prevalence and severity, as well as comorbidities and concomitant medications [46].

All three analyzed antibiotics are widely used for the treatment of severe and nosocomial infections in a hospital setting [15,17,30]; thus, it is expected that healthcare professionals are the main type of reporters for ADRs related to these drugs. Other reasons can explain this distribution, such as healthcare professionals having access to medical information and expertise in the field, the professional and ethical responsibility for patient safety, direct contact with patients, reporting infrastructure access, and awareness. In addition, the relevance of MedDRA terms relevant to drug resistance and their importance for antimicrobial resistance surveillance should be emphasized for healthcare professionals, as well as non-healthcare professionals, to increase the number of submitted reports [47]. 

The percentages of ICSRs containing PTs grouped in the DR category of the total number of ICSRs registered for MER, LIN, and COL were 3.56%, 2.38%, and 8.42%, respectively. We can observe that a large proportion of reports relevant to DR have been recorded for COL, even though they are included in the Reserve group and need to be used as a last-resort treatment option. 

According to the literature, from 2006 to 2012, the use of COL increased almost four times worldwide [48]. In infections produced by multi-drug-resistant gram-negative bacteria, including carbapenem-resistant *Acinetobacter*, *Pseudomonas,* and *Enterobacteriaceae*, COL is sometimes the only antibiotic that is still effective for their treatment [49].

Adverse outcomes related to antimicrobial resistance can result in increased morbidity and mortality. Studies have found substantially higher mortality rates among patients infected with resistant bacteria, such as methicillin-resistant *Staphylococcus aureus*, extended-spectrum beta-lactamase producing *Enterobacteriaceae*, carbapenem-resistant *Enterobacteriaceae*, carbapenem-resistant *Acinetobacter baumannii*, etc., compared to patients infected with susceptible organisms [50]. In our study, we found that between 6 and 24% and between 19 and 35% of the reported ADRs relevant for DR and DI, respectively, had a fatal outcome. 

All three antibiotics showed a higher reporting probability for the analyzed ADRs relevant for DR when compared to MOX, PIP/TAZ, VAN, AMF, and ISA. However, when compared to CEF/AVI, the reporting probability was lower for all three antibiotics.

Regarding ADR relevant for DI, all three antibiotics only showed a higher reporting probability when compared to moxifloxacin.

Nowadays, when antimicrobial resistance is a major problem for public health, continuous research in this field is necessary to complete the level of knowledge. Pharmacovigilance studies are considered triggers for identifying the risk of AMR or the inappropriate use of antibiotics [47]. Accordingly, the present study could be a good instrument for the evaluation of the antimicrobial resistance phenomenon and for highlighting the importance of public health programs for surveillance of the use of antibiotics, and for monitoring AMR or antibiotic ineffectiveness. Moreover, the spontaneous reporting of ADRs is a cost-effective tool used to characterize the safety profile of drugs, based on heterogenous information collected from different countries. Additionally, it might be possible to identify the risks of DR that cannot be observed in pre-authorization studies.

### Limitations of the Study

The statistics available in the EV database only present data on reported ADRs. Because of the nature of spontaneous reporting of ADRs, the results may be influenced by the phenomenon of underreporting or overreporting and reporting bias. The precision of the study might be limited since not all the existing ADR reports associated with drug resistance and drug ineffectiveness of the analyzed antibiotics are available in this database. Moreover, the poor quality of information or the lack of additional useful information in some ICSRs, particularly those related to the patient’s medical condition and other suspected or concomitant administered drugs, may also affect the precision of the results. Thus, the data presented in this study should be interpreted to identify the risk of ADR reporting, rather than to quantify the risk. Moreover, because ICSRs are submitted based on a suspected adverse reaction of a drug and causality is not yet established, data cannot be used to determine and quantify the real risk of ADR occurrence in clinical practice.

## 4. Materials and Methods

### 4.1. Study Design

A retrospective pharmacovigilance study on reported ADRs for COL, MER, and LIN was performed based on spontaneous reports registered in the EV database until 31 December 2022. ICSRs were loaded at https://www.adrreports.eu/portal (accessed on 3 January 2023), the European database of suspected adverse drug reaction reports [37]. ICSRs do not contain patients’ personal information and no ethics committee approval is required [51]. According to European regulations, ICSRs could be completed by healthcare professionals or non-healthcare professionals from the EEA or non-EEA [52]. 

### 4.2. Materials

For each of the three antibiotics (COL, MER, LIN), all ICSRs containing ADRs submitted to EV were considered. These ADRs are codified according to the Medical Dictionary for Regulatory Activities (MedDRA) in more than 25,000 PTs. Based on a protocol published in the literature [47], ADR reports suggesting DR and DI were analyzed. Thus, three PTs relevant to DR, such as “drug resistance”, “multiple-drug resistance”, and “pathogen resistance”, and nine PTs relevant to DI, such as “therapeutic product effect decreased”, “therapeutic product effect incomplete”, “decreased activity”, “drug ineffective for unapproved indication”, “therapeutic product ineffective”, “therapeutic response decreased”, “treatment failure”, “therapy non-responder”, and “drug ineffective” were selected for further analysis. 

### 4.3. Data Analysis

A descriptive analysis was performed to assess the characteristics of all ICSRs reported for MER, LIN, and COL. The criteria used for the analysis included patients’ age, sex, geographical origin, and the category of reporters. Subsequently, all ICSRs related to DR (three PTs) and DI (nine PTs) were extracted, and the fatal ADRs from the total ADRs related to DR and DI were identified. 

According to EMA recommendations, the ROR and 95% CI were calculated for the disproportionality analysis [53]. The equations used for calculating each parameter are presented below [54]:

$$ROR=\frac{a\times d\;}{b\times c}$$

where:

*ROR* = reporting odds ratio
*a* = evaluated ADR for targeted drug
*b* = other ADRs for targeted drug
*c* = evaluated ADR for the drug used for comparison
*d* = other ADRs for the drug used for comparison

95% CI = exp (ln (*ROR*) − 1.96 × SE{ln(*ROR*)}) to exp (ln(*ROR*) + 1.96 × SE{ln(*ROR*)})

where:

CI = confidence interval
SE = standard error

$$SE\left\{ln\left(ROR\right)\right\}=\sqrt{\frac{1}{a}+\frac{1}{b}+\frac{1}{c}+\frac{1}{d}}$$

The data obtained for COL, MER, and LIN were compared with other drugs used in common therapeutic areas and similar clinical contexts. Thus, the same categories of ADRs and PTs reported for other antibacterial drugs (moxifloxacin—MOX, tigecycline—TIG, piperacillin/tazobactam—PIP/TAZ, ceftazidime/avibactam—CEF/AVI, vancomycin—VAN) or antifungal drugs (fluconazole—FLU, isavuconazole—ISA, caspofungin—CAP, amphotericin—AMF, voriconazole—VOR). If the number of ICSRs was less than five or the 95% CI of the ROR was less than 1.0, no signal of disproportionate reporting was assumed [53].

## 5. Conclusions

Antimicrobial resistance is considered a serious threat to public health, especially to patients in the intensive care unit, who are more vulnerable to infections and are frequently on broad-spectrum antibiotic treatment. A retrospective analysis of ADRs reported for meropenem, colistin, and linezolid, which are commonly used to treat infections produced by multi-drug-resistant microorganisms encountered in intensive care unit patients, was performed based on spontaneous reports from the EudraVigilance database. Of the total ADRs reported for each analyzed antibiotic by 31 December 2022, between 2.38 and 8.42% of the reports were related to drug resistance (2.38%—LIN, 3.56%—MER, and 8.42%—COL, respectively), and between 4.15 and 10.14% were related to drug ineffectiveness (4.15%—LIN, 9.45%—MER, and 10.14%—COL, respectively). Moreover, between 6 and 24% and between 19 and 35% of the reported ADRs relevant for DR and DI, respectively, had a fatal outcome. Given the limitations of the spontaneous reporting of ADRs, including the potential for underreporting or overreporting, the results should be interpreted with caution. Our study aims to raise awareness regarding the increasing phenomenon of antibiotic resistance. It also highlights the need to implement surveillance programs to prevent, identify, monitor, and manage ADRs related to drug resistance and ineffectiveness. To achieve good practice in this matter there is a need for personalized dosing strategies, knowledge of patient characteristics, a multidisciplinary approach (critical care physicians, infectious disease physicians, pharmacists, and laboratory staff), and the developing of affordable tools for quantifying the therapeutic response. On the other hand, in order to combat antimicrobial resistance, the health industry should continuously invest in the research and development of new antibiotics or alternative treatment strategies.

## Data Availability

Data are contained within the article.

## Abbreviations

ADR	adverse reaction
AMF	amphotericin
CAP	caspofungin
CEF/AVI	ceftazidime/avibactam
CI	confidence interval
COL	colistin
DI	drug ineffectiveness
DR	drug resistance
EEA	European Economic Area
EMA	European Medicines Agency
EV	EudraVigilance
FLU	fluconazole
ICSR	Individual Case Safety Report
ICU	intensive care unit
ISA	isavuconazole
LIN	linezolid
LPS	lipopolysaccharide
MER	meropenem
MOX	moxifloxacin
PBP	penicillin-binding proteins
PIP/TAZ	piperacillin/tazobactam
PTs	preferred terms
ROR	reporting odds ratio
TIG	tigecycline
VAN	vancomycin
VOR	voriconazole
